# On the radiotoxic ^210^Po in coffee beans worldwide and the impact of roasting and brewing on its extraction into beverages: from the experiments to ^210^Po content prediction

**DOI:** 10.1007/s11356-023-25840-w

**Published:** 2023-02-15

**Authors:** Grzegorz Olszewski, Aleksandra Moniakowska, Dan Zhang, Dagmara Strumińska-Parulska

**Affiliations:** 1 Department of Health, Medicine and Caring Science, Division of Diagnostics and Specialist Medicine, Linköping University, 581 83 Linkoping, Sweden; 2 Environmental Chemistry and Radiochemistry Department, Faculty of Chemistry, University of Gdańsk, 80-308, 63 Gdansk, Wita Stwosza Poland; 3 Institute of Mountain Hazards and Environment, Chinese Academy of Sciences, Chengdu, 610041 China

**Keywords:** Coffee, Extraction, Food products, Diet, Radioactive contamination, Radiation protection

## Abstract

We determined radiotoxic ^210^Po in roasted coffee beans from different regions worldwide, the beverages, and tried to create the prediction model of ^210^Po content based on its growth location. Additionally, the experiments on ^210^Po losses and extraction were performed to describe the actual exposure to ^210^Po. ^210^Po concentrations in coffee beans and brews tuned out low (maximally of 0.20 Bq∙kg^−1^ and 2.31 Bq∙L^−1^, respectively). We assessed the impact of the roasting process on ^210^Po content and its losses at a maximum of 56.7%. During infusion experiments, we estimated the extraction of ^210^Po to the coffee brew at a maximum of 40.6%. The amount of ^210^Po in the coffee brew depended on the infusion style and water type. We calculated the effective radiation doses from the coffee drink ingestion. Coffee drinking does not contribute significantly to the annual effective radiation dose worldwide.

**Graphical Abstract:**

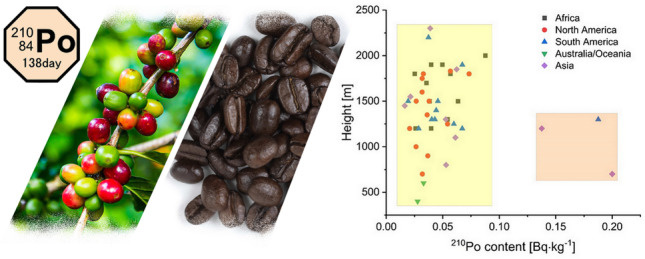

## Introduction

Coffee is a non-alcoholic beverage produced from seeds and berries of the coffee plant (genus *Coffea* L.) (Roselli et al. 2013). More than 100 Coffea genus plants are cultivated in the World. Still, only two species gained substantial economic significance: *Coffea arabica* L. and *Coffea canephora* Pierre ex A. Froeher known as Arabica and Robusta coffee (Alharbi and Alamoudi 2017; Pigozzi et al. 2018). Nowadays, it is one of the most popular and widely consumed beverages globally. The annual consumption varies in different countries, from 12 kg per capita in Finland to 9 kg in Iceland, 6.5 kg in Canada, 5.8 kg in Brazil, 4.2 kg in the USA to 4 kg in Japan and England, and 3 kg in Poland (Chudy 2014; Roselli et al. 2013). *Coffea* trees are cultivated in more than 70 countries, mainly in equatorial Latin America, Southeast Asia, South Asia, and Africa. Unroasted green coffee is one of the most traded agricultural commodities on the international market. The coffee drink is slightly acidic (pH around 5) and can stimulate humans due to its caffeine content. Additionally, it contains minerals, lipids, proteins, fats, carbohydrates, niacin (B3 vitamin), and water (Khandaker et al. 2020; Roselli et al. 2013). Due to its potential to reduce cholesterol levels, lower cardiovascular risks, and lower gastrointestinal and liver cancer risks, coffee is also used in the pharmaceutical, cosmetic, and food industries (Cloete et al. 2019; Romualdo et al. 2019; Khandaker et al. 2020). Green coffee beans are usually roasted, developing the complex flavors that make coffee enjoyable. Chemical reactions that occur during coffee roasting are not well understood. They lead to physical changes in the green beans and the formation of more than 900 volatile compounds, many unknown molecules (melanoidins), and the substances responsible for the coffee beverage’s sensory qualities (Wei and Tanokura 2015).

Like all other vascular plants, the coffee plant uptakes mineral elements and micronutrients from the soil. Accumulation in coffee beans depends on many factors, including species, age, root distribution of the plant, physical and chemical nature of the soil (pH, Eh, organic matter, anions, and cations content), proportions and distributions of elements, the use of fertilizers, and the general climatic conditions (Anderson and Smith 2002). Similar factors govern the uptake of heavy metals and radionuclides in all vascular plants (Olszewski et al. 2019). In the process of metal uptake, the plant root system cannot differentiate between stable and chemically analogous radioactive nuclide. Therefore, potential radiological risks must be considered. Leafy plants can uptake ^210^Po from the soil through the root system and from the air with wet and dry deposition containing short-lived radon daughters (^218^Po, ^214^Pb, ^214^Bi, ^214^Po) and both ^210^Pb and ^210^Po (Persson and Holm 2011).

Polonium ^210^Po, a product of the uranium-radium decay chain, is an alpha emitter with a half-life of 138.38 days, with a specific activity of 166 TBq·g^−1^ (Persson 2014; Persson and Holm 2011). ^210^Po is known as one of the most carcinogenic radionuclides, and its radiotoxic properties gained interest in 2006 after Mr Alexander Litvinienko’s poisoning in the UK. Natural sources of ^210^Po in the environment include uranium ores, deposits, and intense radon fluxes (Puchkova and Bogdanova 2016). An important anthropogenic source of ^210^Po in the natural environment is phosphogypsum storage. As a Technologically Enhanced Naturally Occurring Radioactive material (TENORM), phosphogypsum contains a relatively high ^210^Po activity that can affect local soils biota and water (Boryło et al. 2013; Olszewski et al. 2016a, b, c).

Food and drink consumption is an essential source of internal radiation dose (United Nations Scientific Committee on the Effects of Atomic Radiation 1993). Even though World Health Organization (WHO) defines coffee as a “non-nutritive dietary component,” some studies show coffee may contain toxic chemical impurities such as trace metals, organic pollutants, or toxins (Alves da Silva et al. 2017; Binello et al. 2021; De Toni et al. 2017; Pigozzi et al. 2018; Winiarska-Mieczan et al. 2021). Moreover, its high worldwide consumption and scarce information on the distribution and enrichment of polonium (^210^Po) make this beverage a crucial research subject from a radiation protection point of view.

The hypothesis was the radioactive ^210^Po was present in coffee beans and was extracted to the infusions. Thus, the presented study was divided into sections to complete the most detailed picture of coffee radioactivity from ^210^Po. This study’s first and main aim was to estimate the activity concentrations of ^210^Po in roasted, ready-to-use coffee beans from different locations worldwide. A series of experiments on ^210^Po losses and extraction were performed to lend credence to the entire exposure to ^210^Po present in coffee. We intended to assess the impact of the roasting process on ^210^Po content and its potential losses during the process and prepared infusion experiments to estimate the ^210^Po extraction level to the coffee brew. The final goal was to calculate the effective radiation doses resulting from the coffee drinks ingestion prepared from the analyzed coffee beans. We also hypothesized that ^210^Po content in coffee beans depended on the location of their growth and altitude; based on potential dependence, we planned to create the prediction model for ^210^Po content in coffee beans worldwide.

## Materials and methods

### Coffee samples

To estimate the activity concentrations of ^210^Po and create a prediction model helpful in evaluating ^210^Po content in coffee beans based on the location of the plantation, we chose multiple coffee beans from worldwide locations. The analyzed 46 coffee brands came from 31 countries (five continents), and their plantation details have been presented in Table 1.

**Table 1:** Characteristic of samples of roasted coffee beans

No	Brand name	Continent region	Country	Subregion state	Type	Height (m)
1	Arabica Australia	Australia/Oceania	Australia	New South Wales	Arabica	400
2	Extra Fancy	Australia/Oceania	USA, Hawaii	Kona	Arabica	600
3	Fully Washed B	Africa	Burundi	-	Arabica	1900
4	Kinyovu Profile	Africa	Burundi	Kirimino	Arabica	1800
5	AA Latumba	Africa	Congo	Southern Kivu	Arabica	1900
6	Dijmmah	Africa	Ethiopia	Kaffa	Arabica	1800
7	Sidamo	Africa	Ethiopia	Sidamo	Arabica	2000
8	Mzuzu	Africa	Malawi	-	Arabica	1200
9	AA	Africa	Tanzania	Mount Kilimanjaro	Arabica	1700
10	AA	Africa	Uganda	Rwenzori	Arabica	1900
11	KCFCS	Africa	Uganda	Bugis	Robusta	1500
12	AA	Africa	Zambia	Northern Province	Arabica	1300
13	AA	Africa	Zimbabwe	Caturra	Arabica	1200
14	India Monsooned	Asia	India	Magundi	Robusta	2300
15	Indonesie Wahana Estate	Asia	Indonesia, Java	Lake Mungkur	Arabica	1200
16	Java Jampit A/WP-1X Estates	Asia	Indonesia, Java	Java Sunda	Arabica	1550
17	Sulawesi Toraja Kalossi	Asia	Indonesia, Java	Toraja	Arabica	1450
18	Arabica Sumatra	Asia	Indonesia, Sumatra	Aceh, Lintong	Arabica	1100
19	AA Padauk Washed (Barma)	Asia	Myanmar (Burma)	Paduak	Arabica	1300
20	Mount Everest Supreme	Asia	Nepal	Nuwakot	Arabica	700
21	Robusta Grade 1	Asia	Vietnam	-	Robusta	800
22	Matari	Asia	Yemen	Bani Matar	Arabica	1850
23	AA	South America	Bolivia	San Juan	Arabica	1400
24	Santos NY2 SSFC 17/18	South America	Brazil	Sao Paulo	Arabica	1300
25	Yellow B. Fazenda Da Lagoa	South America	Brazil	Sul de Minas	Arabica	1100
26	South of Minas	South America	Brazil	Sul de Minas	Arabica	1250
27	Pocos de Caldas	South America	Brazil	Minas Gerais	Arabica	1200
28	Excelso	South America	Colombia	Antioquia, Medellin	Arabica	1300
29	Supremo Medellin	South America	Colombia	Villa Maria	Arabica	2200
30	Altura Bio	South America	Ecuador	Loja	Arabica	1500
31	Andes Gold	South America	Peru	Puno Norte	Arabica	1900
32	SHB Pichanaki Grade 1 Bio	South America	Peru	Chanchamayo	Arabica	1500
33	SHG EP Santa Ana Miravalle H1	South America	Salvador	Balsamo	Arabica	1200
34	Antiqua San Juan SCR90	North America	Guatemala	Antigua Valley San Sebastián	Arabica	1850
35	SHB EP Acatenango	North America	Guatemala	Acatenango	Arabica	1800
36	San Rafael Tarrazu SHB RZ	North America	Cost Rica	Tarrazu	Arabica	1600
37	Serrano Superior	North America	Cuba	Sierra Maestra	Arabica	900
38	Altura Lavado Exclusive	North America	Cuba	Granma	Arabica	700
39	AA	North America	Dominican	Barahona	Arabica	1250
40	Genuine Marcala	North America	Honduras	Marcala	Arabica	1500
41	SHG	North America	Honduras	Casitas, Corquin, Copan	Arabica	1750
42	SHG Royal Momotombo	North America	Nicaragua	Nueva Segovia	Arabica	1200
43	Maragogype	North America	Mexico	Chapas	Arabica	1000
44	Chapas Bio	North America	Mexico	Chapas	Arabica	1800
45	Geisha Esmeralda Mico Lot	North America	Panama	Jaramillo	Arabica	1350
46	SHG EP Boquete Indian Baru	North America	Panama	Chiriqui	Arabica	1500

The second part of the research was the roasting experiment which would answer the question about the impact of the roasting process on ^210^Po content and its potential losses during the process. Ten green coffee beans from different countries and continents were chosen (Table 2).

**Table 2:** Green coffee samples for the roasting experiment

No	Brand name	Country	Type
1	-	Rwanda	Arabica
2	Los Arroyos	Guatemala	Arabica
3	Robusta Grade 1	Vietnam	Robusta
4	Arabica Sumatra	Sumatra	Arabica
5	Excelso	Colombia	Arabica
6	Pitalito	Colombia	Arabica
7	Fully Washed B	Burundi	Arabica
8	Maragogype	Mexico	Arabica
9	Dijmmah	Ethiopia	Arabica
10	SHG	Honduras	Arabica
11	Matari	Yemen	Arabica

Another process that impacts ^210^Po content in coffee drinks is its brewing. The infusion experiments were prepared to assess the ^210^Po extraction level of the coffee brew. Some of European’s most popular commercially available grinded and capsuled coffees were chosen (Table 3). For each experiment, only one repetition was performed, and the combined uncertainty (*k* = 1) was calculated.

**Table 3:** Coffee samples for infusion experiments

No	Brand name / Country	Type
1	Astra	Grinded
2	Tchibo Exclusive	Grinded
3	Tchibo Family	Grinded
4	Rwanda	Grinded
5	Sidamo / Ethiopia	Grinded
6	Kenya	Grinded
7	Costa Rica	Grinded
8	Nescafe Dolce Gusto	Grinded, capsules
9	Nescafe Dolce Gusto Grande	Grinded, capsules
10	Café d’Or Espresso Intenso	Grinded, capsules
11	Nescafe Classic	Instant
13	Jacobs Cronat Gold	Instant

### Green coffee beans roasting experiment

The roasting experiment was performed in the laboratory. Green coffee beans were roasted at 220 °C with constant stirring for 10 min. After cooling down, the roasted coffee beans were weighed to check the weight loss. ^210^Po loss during roasting was quantified based on its concentration difference in roasted and unroasted/green coffee beans.

### Infusion experiments

The impact of three types of coffee preparation on the ^210^Po extraction was tested: an overflow coffee maker, a capsule coffee machine, and a French Press preparation. Additionally, three instant coffees were infused with an assumption that all ^210^Po contained in the soluble coffee crystals is dissolved in water and consumed with the beverage. All infusions were prepared using two types of water: tap water and water filtered through a commercially available activated carbon filter with ion exchange resin. The assumption was to investigate if the modification of water chemical composition influences ^210^Po extraction from the coffee. The overflow coffee maker used regular paper filters and water at 96 °C. The standard capsule coffee machine (around 80 °C water temperature and up to 15 bars of pressure) was used to brew coffee drinks from capsules. A French Press coffee was prepared by adding boiling water to grinded coffee beans with stirring for 5 s. All infusions were prepared with tap and filtered water. Additionally, blank water samples for all infusions were prepared to compare the amount of ^210^Po in coffee extracts and clear water used for infusions.

### ^210^Po radiochemical determination and measurement

All samples before the analysis were spiked with a known amount of certified radioanalytical tracer ^209^Po (10 mBq) purchased from the National Physical Laboratory (London, UK). Coffee extracts were evaporated and mineralized using concentrated nitric acid. Coffee beans were digested, and the residue was filtered. Polonium was autodeposited on silver discs from 0.5 M HCl solution with ascorbic acid addition (Flynn 1968; Skwarzec 1997). Nitric acid (65%), hydrochloric acid (37%), and ascorbic acid were obtained from POCH Avantor (Gliwice, Poland). All used chemicals were analytical grade. Pure silver (0.999) was used for Po electrodeposition. ^210^Po was analyzed using an alpha spectrometer (Alpha Analyst S470, Canberra), and the activity concentration of ^210^Po in clear water samples was subtracted from their content in infusions. Due to low ^210^Po concentrations in analyzed samples, the average alpha measurement time was around 7 days. The ^210^Po yield in the analyzed samples ranged from 95 to 99%. The results of ^210^Po activity concentrations were given with expanded combined uncertainty calculated for 95% confidence intervals. The accuracy and precision of the radiochemical method were positively evaluated using IAEA reference materials (IAEA-414; IAEA-384), and both were estimated at less than 5%. The calculated MDA was 0.3 mBq.

### Statistical analysis

For results, discussion series of statistical and chemometric analyses were performed, including the *U* (Mann–Whitney) test, analysis of variance (two-way ANOVA), multiple regression analysis (MLR), and principal component analysis (PCA). All datasets were tested for normal distribution using the Shapiro–Wilk test (*α* = 0.05) and variance homogeneity using the Levene test (*α* = 0.05) before statistical analyses. If necessary, data were normalized. All tests, analyses, and graphs were performed in OriginPro 2022 (OriginLab Corporation, USA).

## Results and discussion

### Coffee beans results

**Fig. 1:**
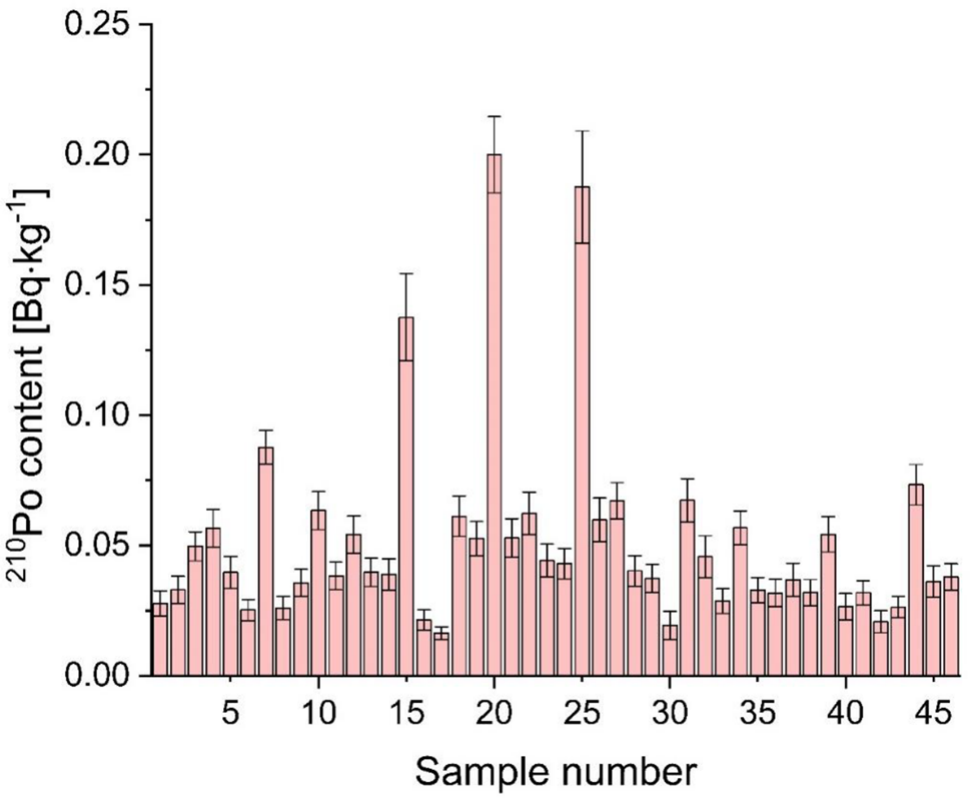
^210^Po concentration in analyzed roasted coffee beans (samples number according to Table 1)

In 46 analyzed brands of roasted coffee beans (Table 1), ^210^Po concentrations ranged from 0.02 ± 0.01 to 0.20 ± 0.01 Bq∙kg^−1^ (Fig. 1). These results are more than ten times lower than ^210^Po and ^210^Pb activities in previously analyzed herbal teas (Moniakowska et al. 2020; Olszewski et al. 2019) and mushrooms (Strumińska-Parulska et al. 2017, 2020, 2021) but similar to activities obtained for different types of farming food products (Strumińska-Parulska and Olszewski 2018). Compared to another naturally occurring radionuclide ^40^ K, which significantly increases an effective radiation dose, ^210^Po results are up to a thousand times lower (Alharbi and Alamoudi 2017; Roselli et al. 2013). The measured ^210^Po content in roasted coffee beans is similar to values reported by other researchers. ^210^Po content in Italian coffee ranges from 0.0336 to 0.114 Bq∙kg^−1^ (Roselli et al. 2013), while in Egyptian Misr coffee and Nescafe, it is lower than 0.01 Bq∙kg^−1^ (Salahel Din 2011).

### Green coffee beans roasting

Coffee roasting may decrease some active compounds, especially volatile ones, being removed at the highest level (Montenegro et al. 2021; Toci et al. 2020). Polonium is a volatile element even in relatively low temperatures, and at a typical roasting temperature of 500–700 °C, all volatile polonium compounds are eliminated (Kovács et al. 2007; Bogdan Skwarzec et al. 2001). Observed ^210^Po loss and mass loss during the green coffee beans roasting process are summarized in Table 4. ^210^Po loss changes substantially (from 10.7 to 56.7%). In contrast, coffee of different brands’ weight loss remains relatively stable (19.4 ± 1.7%). Generally, a roasting degree can be measured by the bean’s color or weight loss. Based on the coffee industry roasting parameters, our roasting process can be identified as medium roasting degree (high roast or full city roast) with weight loss between 14 and 21% (Wei and Tanokura 2015). Medium roasts are performed at temperatures up to 225 °C, corresponding to the 220 °C used in our experiment. In dark roasting degree, a temperature of up to 240 °C is used, while in light roasting, 180 to 205 °C (Sobri et al. 2022). It is seen that ^210^Po loss does not depend on the roasting process but rather on other green coffee beans parameters. Polonium bonds to proteins due to its similarity to sulfur (International Atomic Energy Agency 2017). During the roasting process, the chemical Maillard reaction occurs between reducing carbohydrates and various amino acids, peptides, and proteins containing free amino acids. It leads to protein degradation and denaturation. At the same time, green coffee bean protein subunits integrate into polymeric structures called melanoidins. Different melanoidin compositions in roasted coffee beans suggest several pathways of their formation. Five minutes of roasting lead to the complete disappearance of some free amino acids (Wei and Tanokura 2015). The difference in ^210^Po loss between different coffee brands could be associated with a different chemical composition of the analyzed coffee beans.

**Table 4:** ^210^Po and weight loss of green coffee beans roasting

Brand name	Country	Type	^210^Po loss [%]	Weight loss [%]
-	Rwanda	Arabica	21.9 ± 2.6	18.2
Los Arroyos	Guatemala	Arabica	10.6 ± 2.2	19.3
Robusta Grade 1	Vietnam	Robusta	25.5 ± 4.8	17.8
Arabica Sumatra	Sumatra	Arabica	18.8 ± 3.2	17.9
Excelso	Colombia	Arabica	36.7 ± 6.9	17.4
Pitalito	Colombia	Arabica	19.3 ± 2.6	18.1
Fully Washed B	Burundi	Arabica	34.3 ± 5.1	18.4
Maragogype	Mexico	Arabica	41.1 ± 8.8	20.1
Dijmmah	Ethiopia	Arabica	56.7 ± 11.7	21.3
Sidamo	Ethiopia	Arabica	10.7 ± 1.3	19.2
SHG	Honduras	Arabica	29.1 ± 6.3	21.9
Comsa	Honduras	Arabica	47.9 ± 6.4	20.4
Matari	Yemen	Arabica	34.4 ± 5.9	22.3

### ^210^Po activity concentrations in coffee brews and their extraction efficiency

**Fig. 2:**
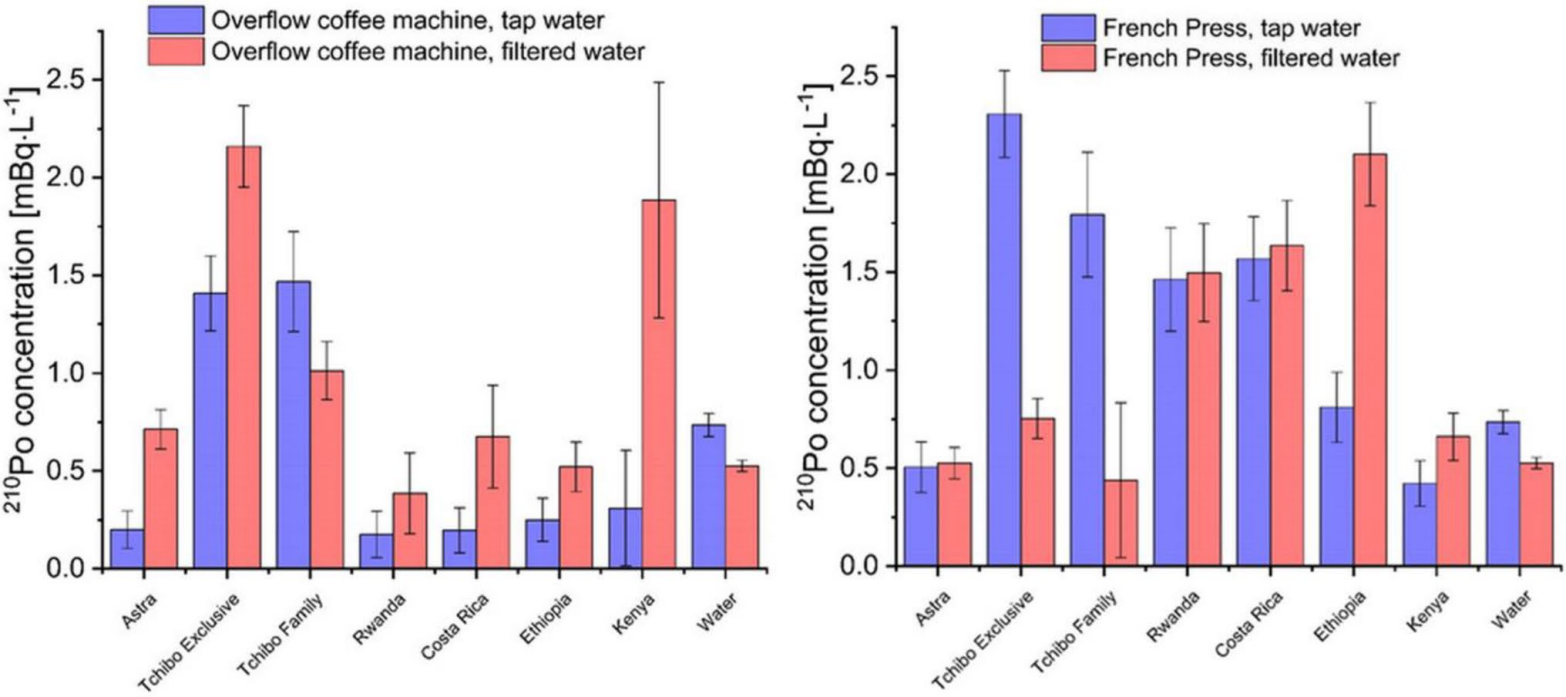
^210^Po concentration in coffee brews from overflow coffee machine and French press

**Fig. 3:**
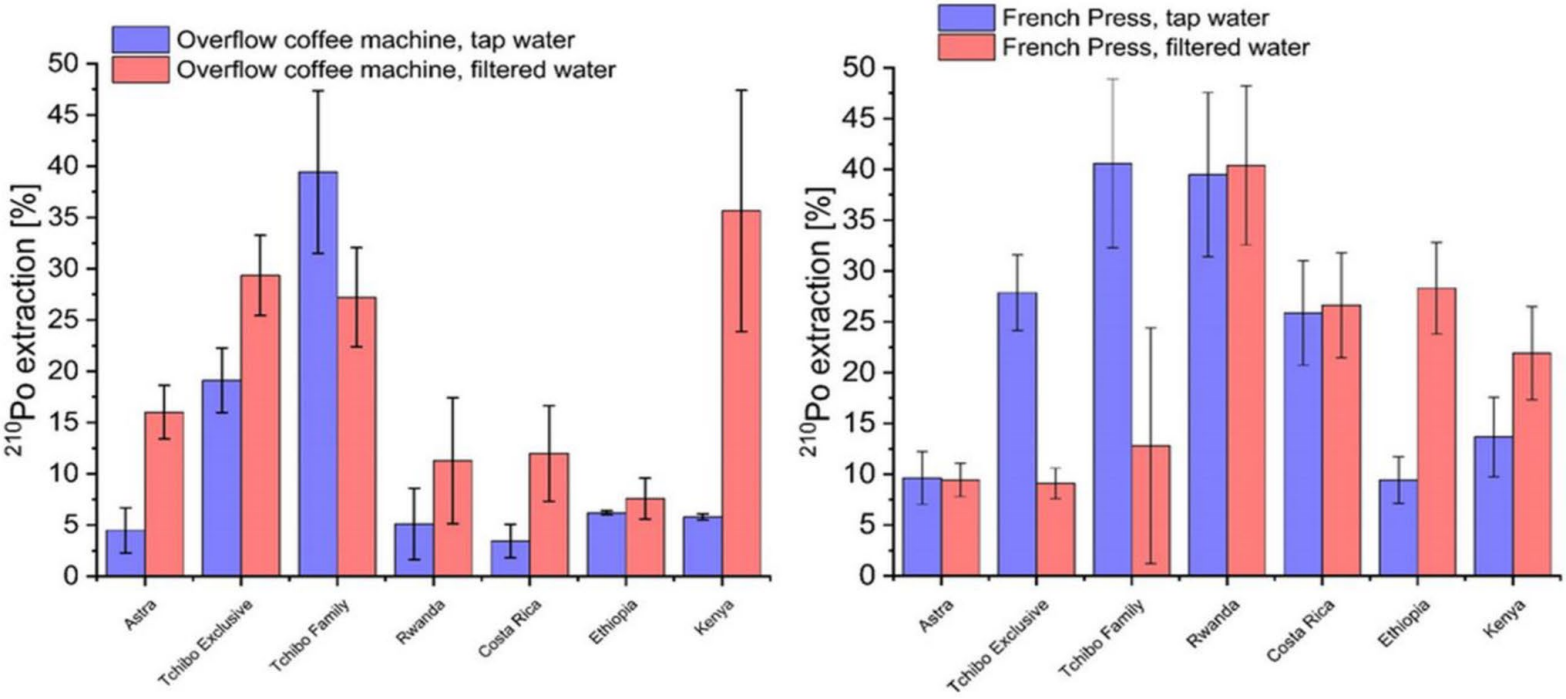
^210^Po extraction efficiency in the coffee brew from overflow coffee machine and French press

^210^Po activity concentrations in coffee brews from an overflow coffee machine and French Press are presented in Fig. 2, while extraction efficiencies are in Fig. 3. In coffee brewed from an overflow coffee machine, ^210^Po concentrations were between 0.2 ± 0.1 and 1.5 ± 0.3 mBq∙L^−1^ for tap water and between 0.4 ± 0.2 and 2.2 ± 0.2 mBq∙L^−1^ for filtered water. Coffees prepared with French Press were characterized by ^210^Po concentrations from 0.4 ± 0.1 to 2.3 ± 0.2 mBq∙L^−1^ for tap water and from 0.4 ± 0.4 to 2.1 ± 0.3 mBq∙L^−1^ for filtered water. The highest ^210^Po concentration was measured in Tchibo Exclusive brew (2.2 ± 0.2 mBq∙L^−1^ for overflow coffee machine, filtered water, and 2.3 ± 0.2 mBq∙L^−1^ for French Press in tap water). These values are significantly lower than concentrations in herbal tea infusions that contained up to 3.9 ± 0.1 Bq∙kg^−1^ of ^210^Po (Olszewski et al. 2019). For Italian coffee brews, ^210^Po concentrations were between 0.7 and 6.7 mBq∙L^−1^ (Roselli et al. 2013). Based on the *U* (Mann–Whitney) test, there are no statistically relevant differences in the ^210^Po concentrations between coffees brewed in tap and filtered water for both overflow coffee machines and French Press (*p* = 0.07 and *p* = 0.79, respectively, for *α* = 0.05). It is worth noticing that *p* = 0.07 is relatively close to the significance level of 0.05. With *α* = 0.1, statistically relevant differences between the two types of water in the overflow coffee machine would exist. When using the French Press, no observed differences are probably connected to a maximum ^210^Po extraction with any water when applying even slight pressure and agitation. The *U* (Mann–Whitney) test was also performed between ^210^Po concentrations in coffee brews from the overflow coffee machine and French Press using tap water. It showed statistically relevant differences (*p* = 0.03 for *α* = 0.05) between these two infusion types. No statistically significant differences were calculated in filtered water between both infusion types (*p* = 1 for *α* = 0.05). In this case, we cannot conclude that water filtering impacts ^210^Po concentration in coffee brews. On the other hand, we can clearly see that using an overflow coffee machine or French Press affects ^210^Po concentrations in the coffee beverage in the case of tap water. The lack of the difference between those two infusion types from filtered water is probably connected to the fact that ^210^Po extraction reaches the maximum in the overflow coffee machine during the constant addition of fresh filtered water to the filter. On the other hand, additional slight pressure and agitation in French Press with the filtered water does not increase ^210^Po extraction to the beverage.

Calculated ^210^Po extraction efficiencies for coffee brewed from an overflowing coffee machine were between 4.5 ± 0.2 and 39.4 ± 0.5% for tap water and between 7.5 ± 0.1 and 35.7 ± 1.3% for filtered water. Coffees prepared with French Press were characterized by ^210^Po extraction efficiency from 9.4 ± 0.1 to 40.6 ± 0.5% for tap water and from 9.1 ± 0.1 to 40.4 ± 0.5% for filtered water. Around 40% ^210^Po extraction efficiency into the coffee brew was measured for Tchibo Family coffee, in both an overflow coffee machine and French Press from tap water. These values are similar to ^210^Po extraction efficiencies obtained for Italian coffees (between 11 and 33%) (Roselli et al. 2013) and herbal tea infusions (between 7.5 and 27.4%) (Olszewski et al. 2019). Extraction intensity strongly depends on the solubility of the compound. They could be either strongly bound to the matrix or more soluble in the solution. Polonium can form organic compounds with polyphenols in high temperatures, influencing its extraction level to the infusion (Puchkova and Bogdanova 2016).

Separate tests were performed for capsule coffee machines and instant coffee. Three types of capsules were used, and the results are presented in Table 5. A capsule coffee machine is a high-pressure coffee maker that ensures a high-quality coffee aroma. Calculated ^210^Po concentrations in capsule coffee drinks (Table 5) are comparable to other types of coffee infusion (Fig. 2), though ^210^Po extraction efficiency does not reach the highest values of different infusion types (Fig. 3). Performed *U* (Mann–Whitney) test shows no statistically relevant differences between ^210^Po concentration in coffee brew prepared by capsule coffee machine with tap and filtered water (*p* = 0.4 for *α* = 0.05).

**Table 5:** Results for the capsule coffee machine

Brand name	^210^Po content (Bq∙kg^−1^)	^210^Po concentration (mBq∙L^−1^)		^210^Po extraction (%)		Annual dose (nSv)*	
		Tap water	Filtered water	Tap water	Filtered water	Tap water	Filtered water
Nescafe Dolce Gusto Lungo	0.11 ± 0.01	0.17 ± 0.12	0.24 ± 0.07	4.35 ± 0.09	6.09 ± 0.03	23	33
Nescafe Dolce Gusto Grande	0.19 ± 0.01	0.65 ± 0.13	1.16 ± 0.16	8.05 ± 0.03	14.25 ± 0.04	88	156
Café d’Or Espresso Intenso	0.09 ± 0.01	0.23 ± 0.13	0.43 ± 0.14	6.36 ± 0.13	12.84 ± 0.16	30	58

^*^Assuming two coffee consumption per day

Additionally, two types of instant coffees were tested, and the results are presented in Table 6. ^210^Po content in both brands was low compared to analyzed coffee beans (Fig. 1). This can be explained by instant coffee being used to be produced from an extract prepared from roasted coffee beans. Therefore, ^210^Po content is significantly different from roasted coffee beans (Fig. 1). At the same time, calculated ^210^Po concentrations for coffee drinks prepared from both brands (assuming that 100% of the ^210^Po retained in the instant coffee crystals are extracted to the beverage) are similar to those prepared from roasted coffee beans (Fig. 2).

**Table 6:** Results for instant coffees

Brand name	^210^Po content (Bq∙kg^−1^)	^210^Po concentration (mBq∙L^−1^)	Annual dose (nSv)*
Nescafe Classic	0.033 ± 0.002	1.33 ± 0.08	233
Jacobs Cronat Gold	0.018 ± 0.002	0.70 ± 0.09	123

^*^Assuming two coffee consumption per day

### Annual doses due to coffee brews consumption

**Fig. 4:**
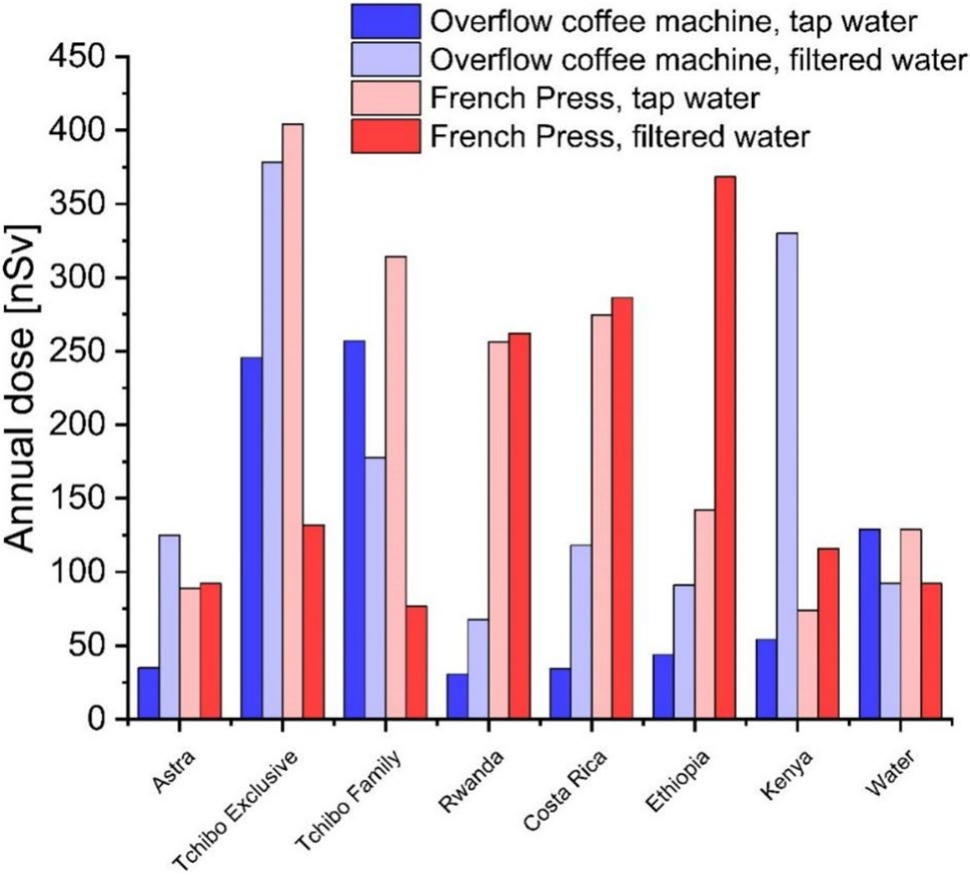
The values of the annual effective dose from ^210^Po ingestion

To identify the potential radiotoxicity of analyzed coffee beverages, the annual effective radiation doses were calculated based on previously calculated ^210^Po activity concentrations (Fig. 4). The effective dose conversion coefficient from ^210^Po ingestion for adult public members recommended by ICRP is 1.2 μSv∙Bq^−1^ (ICRP 2012). The calculation was based on the assumption that a statistical coffee consumer drinks two coffees per day prepared from 10 g of coffee powder in 200 mL of water. All calculated effective doses were given after subtracting the dose-related to water (Fig. 4). The highest annual effective radiation dose from ^210^Po ingestion with coffee beverage was calculated for Tchibo Exclusive (404 nSv for beverage prepared from tap water using French Press, and 378 nSv for beverage prepared from filtered water using overflow coffee machine). The lowest doses (31 and 34 nSv, respectively) were calculated for coffees from Rwanda and Costa Rica prepared from tap water using an overflow coffee machine. In general, we can see the trend of annual dose increase when using filtered water instead of tap water, which is related to the statistically higher ^210^Po extraction to the beverage when using filtered water or French Press. Compared to an all-natural total annual effective dose in Poland (2.1–2.6 mSv/year), calculated doses from drinking coffee beverages are irrelevant from the radiation protection point of view (Pietrzak-Flis et al. 1997). Moreover, computed doses from coffee consumption are significantly lower than annual effective doses from other food products available in Poland (Olszewski et al. 2019; Strumińska-Parulska et al. 2016, 2017, 2021; Strumińska-Parulska and Olszewski 2018). A typical Polish person receives an annual effective dose of between 4.7 and 7.0 µSv from ^210^Po by consuming 116 kg of fruits, vegetables, pasta, and cereals (Strumińska-Parulska and Olszewski 2018). Annual consumption of around 0.5 kg of dry mushrooms (*Macrolepiota procera*, *Leccinum aurantiacum*, and *Boletus edulis*) could lead to radiation dose from ^210^Po from 0.45 to 10.0 µSv (Strumińska-Parulska et al. 2016, 2017, 2021). On the other hand, drinking one herbal tea daily leads to an annual effective dose of ^210^Po between 0.18 and 3.42 µSv (Olszewski et al. 2019). All obtained data showed no radiological risk connected to ^210^Po ingestion with analyzed coffee beverages. The total all-natural annual effective dose in Poland is relatively low compared to the rest of the world; therefore, analyzed coffee brews are radiologically safe worldwide.

### Chemometric analysis and ^210^Po content prediction in coffee beans worldwide

Chosen coffee beans came from five continents and 31 countries. Their plantations were localized at different heights above sea level (Table 1). We hypothesized these attributes available for all consumers could be used in predicting ^210^Po concentration, assuming its accumulation is directly connected with the natural radioactivity of soil and the amount of wet and dry deposition. It is known that ^210^Po and ^210^Pb deposition varies with the latitude, longitude, and height above sea level (Persson 2014). It is possible to predict ^210^Pb activity and ^210^Po/^210^Pb activity ratio based on longitude, latitude, and height above the sea level using PLS regression. Such models were created based on multiple data from different locations worldwide (Persson 2016). It is also recognized that trace metal composition reflects the mineral composition of the soil and the plant’s environment (Anderson and Smith 2002). Regular coffee consumers purchase packed roasted coffee beans with precise information to localize most of these parameters, at least up to the continent, country, and sometimes region of the plantation. Still, any additional information about the plant (stable elements concentration, organic compounds concentrations etc.) creates a higher probability of making a robust prediction model. Chemical profiling is common in food chemistry, especially to differentiate the growing origins. This practice was also employed for coffee beans using stable elements, volatiles, and a series of chemometric analyses (Anderson and Smith 2002; Demianová et al. 2022; Habte et al. 2016). In our case, a simplified synthetic approach was chosen to find a multivariate correlation between ^210^Po concentration and coffee growing location only by using the information provided by the coffee producers. Knowing the location of the coffee plantation and its height above sea level, we looked for a relationship between seemingly uncorrelated data and tried to create a prediction model for ^210^Po concentration. Multiple regression analysis was performed on normalized data, and the results showed that this kind of prediction only based on the continent and height above the sea level is not possible (for *α* = 0.05 *p* = 0.14 for intercept; *p* = 0.22 for height; *p* = 0.25 for Africa, *p* = 0.42 for N. America, *p* = 0.15 for S. America; *p* = 0.07 for Asia; Australia/Oceania was excluded due to the low number of results). The fitting function is not significantly better than the constant function at the assumed significance level. Similar results were obtained using two-way ANOVA (for *α* = 0.05 *p* = 0.18 for continent and *p* = 0.89 for height), which means no statistically relevant differences in ^210^Po concentration in coffee beans between analyzed continents and altitude. Similar results were observed in *Wolfiporia cocos* mushrooms from China, where no correlation between ^210^Po concentration and height above sea level was noticed (Strumińska-Parulska et al. 2022).

**Fig. 5:**
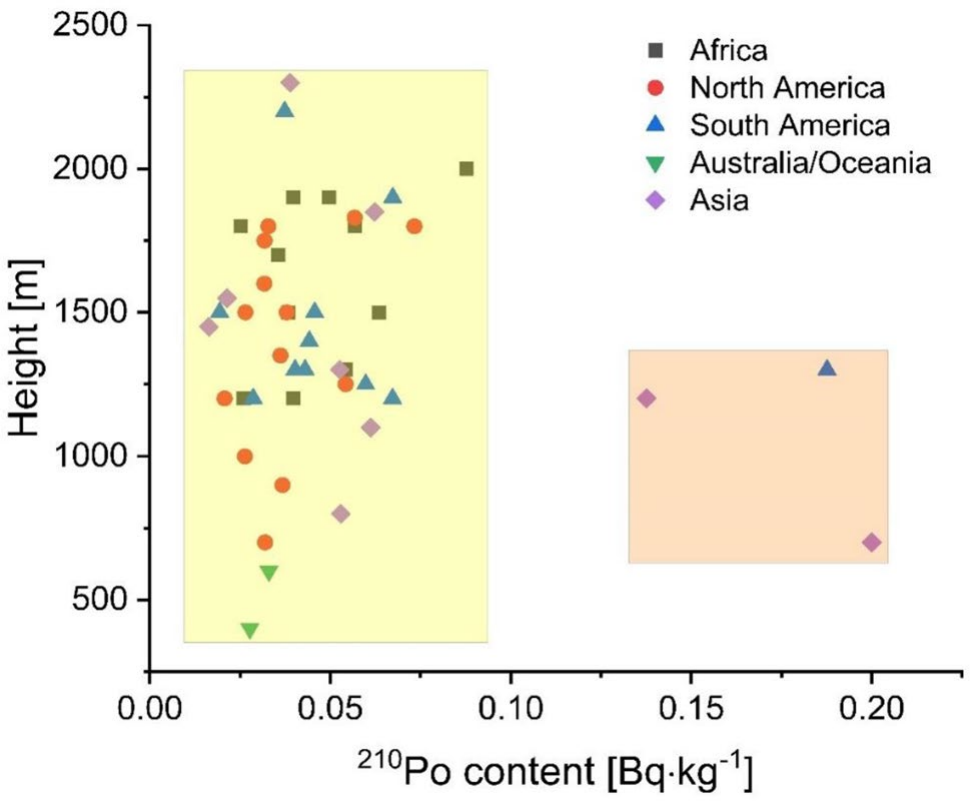
Relation between height above the sea level, ^210^Po concentration in coffee beans and continents

This pattern is projected in Fig. 5, where the relation between height above sea level and ^210^Po concentration in roasted coffee beans are estimated in samples from different continents. Calculated Pearson’s *r* value is − 0.11, suggesting a statistically irrelevant negative correlation between ^210^Po concentration in coffee beans with plantation height (with *p* = 0.43 at *α* = 0.05, the slope is not significantly different from zero). In Fig. 5, we can distinguish two groups of samples, and the relation between the continents is not seen. Three samples are characterized with ^210^Po content higher than 0.1 Bq∙kg^−1^ (two samples from Asia and one from South America). There is no apparent connection between those three samples, suggesting that other, more critical factors govern the uptake of ^210^Po to the coffee plants.

**Fig. 6:**
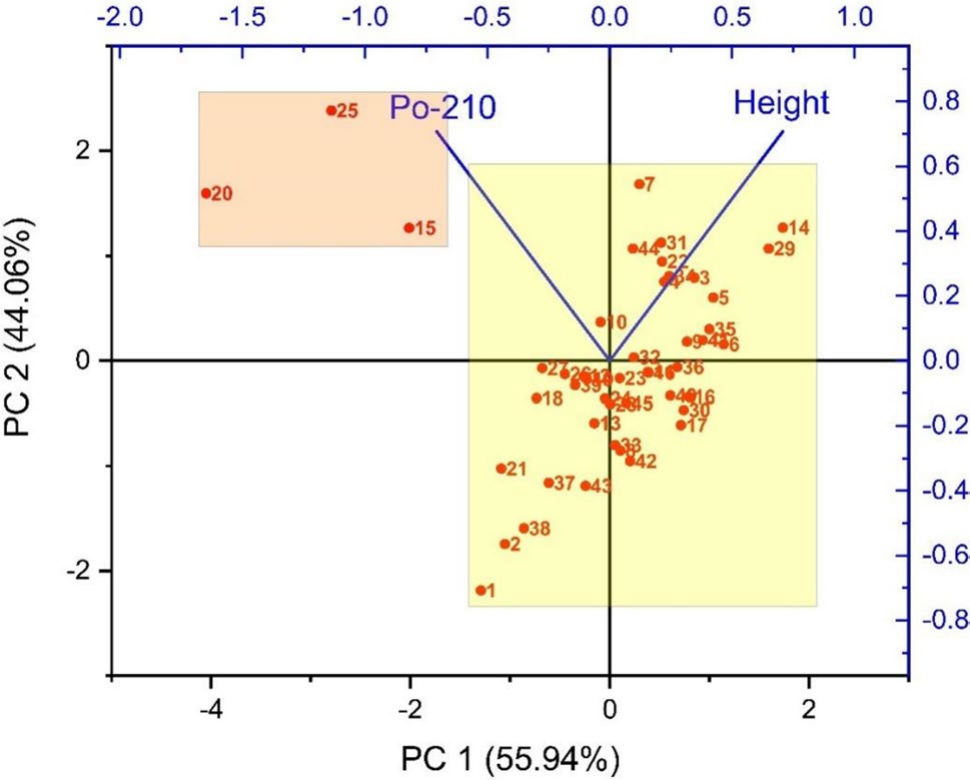
PCA results for coffee samples analyses

The PCA analysis confirms these results. The presented biplot (Fig. 6) similarly shows two groups of samples, one marked in yellow related to height loading and marked in orange to ^210^Po loading. We can see an apparent relation between most samples in the yellow box and height loading. When the three outliers from the orange box are removed, Pearson’s *r* factor changed from − 0.11 to 0.24, suggesting a statistically irrelevant positive correlation between ^210^Po concentration in coffee beans with plantation height (with *p* = 0.11 at *α* = 0.05). ^210^Po activity in the soil, which is mainly connected with the specific natural geological background, as well as the amount of wet and dry precipitation, can vary over a short distance. Therefore, the hypothesis that ^210^Po content in coffee beans depended on the location of their growth and the altitude was not fulfilled, and it is impossible to correlate ^210^Po concentration in coffee beans with height above sea level and the continent of the plantation only.

## Conclusions

Calculated ^210^Po content in coffee beans and ^210^Po concentrations in coffee brews are relatively low, which is connected to scarce ^210^Po accumulation in coffee beans. ^210^Po was extracted to coffee brew with an efficiency between 4.5 and 40.6%, and the roasting process removed up to 56.7% of the volatile forms of ^210^Po. We confirmed that using filtered water for coffee brewing slightly increases ^210^Po concentrations in the brew from the overflow coffee machine. At the same time, we observed that higher ^210^Po concentrations in coffee are present when tap water is used in a French Press instead of an overflow coffee machine. We can conclude that water and infusion type are important factors governing ^210^Po extraction from coffee beans. Calculated annual effective doses from prepared coffee brews are significantly lower than for other food products in Poland and are radiologically safe. The study showed that by only using the information provided on the coffee package (continent, altitude), it was impossible to predict ^210^Po concentration in roasted coffee beans and develop a working model. Even though most producers provide a region name where the plantation is located, the typical coffee consumer cannot evaluate the order of magnitude of ^210^Po content.

## Data Availability

Data available on request from the authors — the raw data supporting this study’s findings are available from the corresponding author upon reasonable request.
